# Who Takes Precautionary Action in the Face of the New H1N1 Influenza? Prediction of Who Collects a Free Hand Sanitizer Using a Health Behavior Model

**DOI:** 10.1371/journal.pone.0022130

**Published:** 2011-07-15

**Authors:** Tabea Reuter, Britta Renner

**Affiliations:** Psychological Assessment and Health Psychology, Department of Psychology, University of Konstanz , Konstanz, Baden-Württemberg, Germany; University of Hong Kong, Hong Kong

## Abstract

**Background:**

In order to fight the spread of the novel H1N1 influenza, health authorities worldwide called for a change in hygiene behavior. Within a longitudinal study, we examined who collected a free bottle of hand sanitizer towards the end of the first swine flu pandemic wave in December 2009.

**Methods:**

629 participants took part in a longitudinal study assessing perceived likelihood and severity of an H1N1 infection, and H1N1 influenza related negative affect (i.e., feelings of threat, concern, and worry) at T1 (October 2009, week 43–44) and T2 (December 2009, week 51–52). Importantly, all participants received a voucher for a bottle of hand sanitizer at T2 which could be redeemed in a university office newly established for this occasion at T3 (ranging between 1–4 days after T2).

**Results:**

Both a sequential longitudinal model (M2) as well as a change score model (M3) showed that greater perceived likelihood and severity at T1 (M2) or changes in perceived likelihood and severity between T1 and T2 (M3) did not directly drive protective behavior (T3), but showed a significant indirect impact on behavior through H1N1 influenza related negative affect. Specifically, increases in perceived likelihood (β = .12), severity (β = .24) and their interaction (β = .13) were associated with a more pronounced change in negative affect (M3). The more threatened, concerned and worried people felt (T2), the more likely they were to redeem the voucher at T3 (OR = 1.20).

**Conclusions:**

Affective components need to be considered in health behavior models. Perceived likelihood and severity of an influenza infection represent necessary but not sufficient self-referential knowledge for paving the way for preventive behaviors.

## Introduction

On 12 April, 2009, the Government of Mexico responded to a request by the World Health Organization (WHO) for verification of an outbreak of acute respiratory infections in the small rural community of La Gloria, Veracruz. From 22–24 April, 2009, a new influenza A (H1N1) virus infection, commonly called “swine flu”, was confirmed in several patients. On 11 June, 2009, the 2009 (H1N1) pandemic was declared by the WHO. All countries were advised to be on high alert and to strengthen infection control measures in health facilities. As of October 23, 2009, when the present study was launched, there have been more than 414,000 laboratory-confirmed cases of pandemic influenza (H1N1) worldwide and nearly 5,000 deaths were reported to WHO [1]. As a consequence, during the winter of 2009, fears rose that a second wave of the pandemic spread would occur, and many countries were planning national prevention campaigns on the basis of WHO safety recommendations. In addition to getting vaccinated against H1N1, the WHO mainly recommended behavior-related preventive measures. Specifically, they recommended that people keep at least one meter distance from people showing symptoms of influenza-like illness, reduce the time spent in crowded settings, improve airflow in living spaces by opening windows, avoid touching their mouth, nose and eyes when possible, and most importantly, regularly clean their hands thoroughly with soap and water or an alcohol-based hand rub [2].

### Cognitive and Affective Risk Perceptions

However, from a psychological perspective, the presence of an actual health risk such as an emerging influenza pandemic is not sufficient to trigger preventive behavior such as regular and proper cleaning of one's hands. Almost all health behavior theories assume that people need to feel personally at risk in order to take protective action [3]–[6]. In most common health behavior models, the belief that one might be in danger is commonly defined as ‘perceived risk’ which encompasses two aspects: (a) the perceived probability of getting influenza and, (b) the perceived seriousness of an influenza infection. Formally, perceived risk should be proportional to both, the perceived probability and severity of an influenza infection (risk = probability×severity of the influenza) which implies that greater likelihood of infection and severity result in a greater overall perceived risk [6], [7]. Accordingly, the greater the numbers of perceived risk, the more likely people should be to take protective action (motivational hypothesis; [6], [8]). Supporting this notion, a meta-analysis of longitudinal studies on the relation between perceived risk and vaccination behavior yielded a mean effect size of *r* = .29 for perceived probability and vaccination and *r* = .23 for perceived severity and vaccination [8].

However, recent conceptions of risk have stressed the importance of more affect-related aspects of risk perception [9], [10]. Peters and Slovic [11] found that the psychological dimensions of risk can be distilled into two primary factors: dread, defined by the extent of perceived lack of control, feelings of dread, and perceived catastrophic potential; and risk of the unknown, the extent to which the hazard is judged to be unobservable, unknown, new, or delayed in producing harmful impacts. The first of these dimensions clearly suggests an affective rather than cognitive evaluation of hazards. Accordingly, Slovic and colleagues [10], [12] have proposed an “affect heuristic” assuming that people rely on general affective evaluations in making risk and benefit judgments which highlights the importance of affect for risk perceptions and risk-related behavior. In a similar vein, the “risk-as-feelings” hypothesis proposed by Loewenstein et al. [9] assumes that responses to risky situations result in part from emotional or affective influences, including feelings such as worry, fear, or threat. The idea that negative affect-related facets of risk perceptions may facilitate protective behavior has received repeated support in the context of cancer screening behaviors. A meta-analysis showed that breast cancer worry, one form of affect-related risk perception, predicts reliably a greater likelihood of screening [13], [14].

These two conceptualizations of risk raise the question about the relative importance of numerical-cognitive (e.g., probability or severity beliefs) and affect-related facets of risk perception (e.g., experienced worry, threat, or fear) in the prediction of protective behaviors. To date, research has yielded mixed results with some studies only demonstrating a predictive value for either numerical-cognitive [15] or affect-related risk perceptions [16], while others demonstrated an effect for both [17]–[20] (see [21] for a review). Instead of assuming either a direct impact of numerical-cognitive or affect-related risk perception on protective behaviors, the risk-as-feelings conception [9] integrates both perspectives by assuming that affect mediates, at least in part, the relationship between an individual's cognitive evaluation of risk and his or her behavioral response to it. Thus, numerical-cognitive risk perceptions might exert an indirect effect on protective behavior through affect-related risk perceptions [17], [22], [23].

Moreover, previous studies predominately examined the relative impact of both facets of risk perceptions within the context of commonly well-known health threats (e.g., cancer, flu infection). These studies might have been only of limited informative value since the respective hazard was no longer associated with an immediate threat and urgency for taking precautions. Thus, people might have already digested the risk, resulting in comparably stable and affectively “cooled-off” risk perceptions and beliefs about precautions. For example, most people know that smoking is bad for their health and that taking a flu shot might be a reasonable protective measure but the affective significance of the hazard might be often rather small due to habituation effects [24]. Conversely, when people are confronted with a new hazard, such as the H1N1 pandemic in 2009, they need to gauge the immediate threat as well as the need to take new precautions in order to protect themselves. Renner, Schüz, and Sniehotta [25] showed, in the context of the outbreaks of the BSE (“mad cow disease”) and FMD (Foot and Mouth Disease) epidemics in Germany, in 2001, evidence for heightened risk perceptions and interest in behaviors change at the beginning of the livestock epidemic. In a similar vein, Jones and Salathé [26] observed, in their cross-sectional online survey within an US sample, that higher self-reported anxiety over the H1N1 influenza pandemic was related to protective behavior at the beginning of the survey. Thus, new hazards and new possibilities for precaution are more likely to prompt people to gauge their risk and to consider changing their behavior [6], [25], [27] in a relative affective or “hot” mind set; thereby, allowing a more straightforward examining of the interplay between numerical-cognitive and affect-related risk perceptions.

### Risk Perceptions – Preventive Behavior: A Longitudinal Perspective

Most studies to date have examined cross-sectional relations between risk perceptions and preventive behaviors [6]. However, interpreting cross-sectional data is notoriously difficult since they confound motivational and accuracy related aspects of the relation between risk perceptions and preventive behavior [28]. Specifically, the motivational hypothesis assumes a positive correlation between both variables indicating that high perceived risk leads people to act. In contrast, the accuracy hypothesis predicts a negative relationship between risk perception and preventive behavior since people who behave in a more risky way should also feel more at risk. Accordingly, a negative correlation indicates relative accurate risk perceptions: people are aware of their risk status [6], [25], [27], [28]. Consequently, for studying the positive motivational effect of risk perceptions on preventive behavior, a longitudinal research design is required demonstrating that higher risk perceptions subsequently lead to more protective behavior [6], [28]. Moreover, to study the idea that numerical-cognitive risk perceptions exert an indirect effect on protective behavior through affect-related risk perceptions, a longitudinal design with three measurement points of time is needed.

### The Present Study

The present study aims to examine how numerical-cognitive and affect-related risk perceptions are related to precautionary behavior in the context of a newly emerging H1N1-virus (swine flu) pandemic. In September 2009, the first serious case of swine flu occurred in Germany with a 35 year old man being hospitalized in intensive care. When the present study was launched in October 2009, the vaccine became available to the public (in week 44) and the first increase in fatalities occurred [29]. By the end of November (week 47), the number of fatal cases rose to a maximum of 37 [29] with more than 46,000 cases being reported in one week [30]. During that time, the German media drew a grim future, warning against a new flu epidemic which might claim a high death toll among the German population. German health authorities, e.g., the Robert Koch Institute, issued warnings and asked the public to comply with WHO safety recommendations. In particular, cleaning one's hands thoroughly with soap and water or with an alcohol-based hand rub on a regular basis was heavily promoted. Accordingly, hand sanitizer came into public focus as a cost-effective and safe possibility for decreasing one's infection risk. From the end of December 2009 till January 2010, week 52 of 2009 to week 1 of 2010, a second peak in fatal cases occurred with 20 fatalities [29]. In a longitudinal study, we tested whether this newly promoted protective measure was driven by numerical-cognitive and/or affect-related H1N1 risk perceptions. Thus, our study examines the rare coincidence of a new hazard and a new precaution measure observed in a controlled environment.

Specifically, we examined who collected a free bottle of hand sanitizer and/or leaflets during the swine flu pandemic in December 2009 (Time 3, T3) after they had taken part in a longitudinal online-survey assessing both forms of risk perceptions in October 2009 (Time 1, T1) and eight weeks later in December 2009 (Time 2, T2). In particular, three different longitudinal models were tested. The *Parallel-Impact Model* (Model 1) tested whether the numerical-cognitive risk perceptions (perceived likelihood, perceived severity, likelihood×severity interaction; T2) and affect-related risk perceptions (perceived threat, worry, concern; T2) independently predicted the hand sanitary pick-up rate measured at a subsequent time point (T3). In two additional models, whether numerical-cognitive risk perceptions exerted an indirect effect on protective behavior through affect-related risk perceptions as proposed by the risk-as feelings-model was tested. The *Time-Sequence Model* (Model 2) examined, within a time sequence, whether cognitive-numerical risk perceptions (T1) impacted subsequent affect-related risk perceptions (T2) which in turn increased the pick-up rate (T3). Accordingly, cognitive-numerical risk perceptions preceded affect-related risk perceptions and the latter preceded the observed preventive behavior. One could argue that Model 2, although realizing a sequential longitudinal design, is still a rather static conception. Therefore, the static time-sequence model was extended by a more dynamic perspective. The *Dynamic-Change Model* (Model 3) examined whether changes in cognitive-numerical risk perceptions were associated with changes in affect-related risk perceptions between T1 and T2 and whether these changes predicted the hand sanitary pick-up rate at T3.

## Methods

### Ethics Statement

The research presented here was approved by the Research Council of the University of Konstanz (AFF 02/10). At the beginning of the questionnaire, a brief introduction about the study aims was given. Respondents were informed that participation in the study is voluntary, that they can withdraw from the study at any time, and that there are no consequences for withdrawing.

### Participants and Procedure

In close temporal coincidence with the first increase of the swine flu epidemic in Germany in October 2009 (weeks 43–44), 646 participants completed an online-questionnaire (Unipark survey software) at Time 1 assessing sociodemographic variables and H1N1-related numerical-cognitive and affect-related risk perceptions. The questionnaire and further study information are available from the authors. Using the snowball technique, participants were invited to the study via an E-mail sent to the student and employee register of the university (students *N* = 9.270; employees *N* = 2.155) and via an offical press release by the university (cf. [26], [31]–[33] for similar a procedure and similar response rates). Eight weeks after the first assessment, 68% (*n* = 439) of the particpants filled in the second questionnaire in December 2009 (week 51–52) assessing again both types of risk perceptions (Time 2; T2). The invitation to the T2 questionnaire included a link to the questionnaire and a voucher for a bottle of hand sanitizer and leaflets which could be redeemed within the next four days at a university office newly established for this occasion. The leaflets contained information on the H1N1 influenza vaccination and recommendations for preventive measures and were published by the Robert Koch Institute. Participants with missing values on variables at T1 or T2 (*n* = 17) were not included in further data analysis resulting in final sample size of *N* = 629 at T1 and *n* = 429 at T2 (32% drop-out).

Of the 629 participants taking part in the first survey at T1, 390 (62%) were women (*M* = 26 years, *SD* = 9.0) and 92% had a high school degree (‘Abitur’). Of these, 19 participants (3.0%) reported that they were vaccinated against H1N1 and 13 reported that they were diagnosed with H1N1 (2.1%). The study sample did not differ significantly in terms of risk perceptions, sex, or education from the drop-out sample. The only difference found indicated that the study sample was two years older than the drop-out sample (*M* = 25, *SD* = 7.1, *t*(627) = 2.76, *p*<.05).

### Measures

#### Numerical-cognitive risk perception

In accordance with previous research [8], [20], perceived absolute and comparative likelihood of an H1N1 infection was assessed by asking participants to estimate (a) their absolute likelihood of getting the H1N1 influenza on a seven-point scale ranging from 1 *(very unlikely)* to 7 *(very likely)* and (b) to estimate their likelihood of getting the H1N1 influenza in comparison to an average peer of their same age and sex on a seven-point rating scale ranging from 1 *(much below average)* to 7 *(much above average)*, Cronbach's α = .61 at T1 and Cronbach's α = .64 at T2. The two items were summed up for a perceived likelihood of infection score. Furthermore, participants were asked how severe they thought a H1N1 influenza would be with response options ranging between 1 *(not serious/can be neglected)*, 4 *(relatively serious, requires sick leave)* and 7 *(very serious/life threatening*) (see also [34]).

#### Affect-related risk perception

Participants provided three different affective risk ratings (cf., [35], [36]. They were asked whether they feel concerned about becoming infected with the H1N1 virus and whether they feel threatened by the H1N1 influenza with the item stem “I feel threatened/I am concerned …”. Answers ranged from 1 (*completely disagree*) to 4 (*completely agree*). Additionally, they were asked how worried they are about their health due to the swine flu with 1 *(not at all worried)* and 7 *(very worried)*. Internal consistency of the three affective risk perception items was high with Cronbach's α = .85 at T1 and Cronbach's α = .81 at T2. All three variables were z-standardized before they were summed up for an affect-related risk perception index.

### Analytical Procedure

In a first step, mean level changes in both types of risk perceptions were analyzed using MANOVA for repeated measures. In descriptive analyses, MANOVAs, and indirect effects missing values were treated by listwise deletion. Therefore, coefficients are based on a varying number of cases. Control analyses were additionally conducted based on listwise deletion across all variables in order to test for systematic dropout biases. In a second step, the main study hypotheses were tested with three different path models employing logistic regression analysis using Mplus Version 5.2. The pick-up rate of hand sanitizer served as dependent variable with, 1 = pick up and 0 = no pick-up. For estimating indirect effects for perceived likelihood and perceived severity, a non-parametric bootstrapping approach was employed [37]. The present analyses are based on a new mediation approach [38], [39], using bootstrapping, which does not require that X and Y are directly associated. In order to differentiate between the two approaches, we use the term ‘indirect effects’ [38] for results based on bootstrapping. Logistic regression coefficients for indirect effects were estimated using a Newton-Raphson iteration algorithm [37]. The variables were centered to render first order effects interpretable when testing the interactions [40]. For path models, full information maximum likelihood estimation (FIML; [41]) was employed to derive parameter estimates in the face of missing data; FIML makes use of all available data in model estimation.

## Results

### Participant Characteristics

In total, 55 (39 women, 16 men) out of the 629 participants redeemed their voucher for a bottle of hand sanitizer and some leaflets in a university office established for this event. The hand sanitizer and leaflets were pick-up by the participants within one to four days following T2. Specifically, 68.1% redeemed the voucher at T2+1day, 25.5% at T2+2 days, 3.6% at T2+3days, and 9.1% at T2+4days. Table 1 summarizes the baseline characteristics of the participants as a function of the pick-up behavior. As Table 1 shows, the majority of participants who came to pick-up the free sample of hand sanitizer, held a university degree which was to be expected due to the study setting. However, the proportion of participants without a university degree was significantly higher in the group who came to pick-up the hand sanitizer sample. Participants under 21 years of age were less likely to redeem the voucher than participants over the age of 25 years. No differences in the pick-up behavior in dependence of gender, diagnosed H1N1 infection or vaccination status were observed.

**Table 1:** Baseline characteristics of participants as a function of the pick-up behavior (a free sample of hand sanitizer and leaflets).

	Participants who picked-up the free sample of hand sanitizer, n (%)	Participants who did not pick-up the free sample of hand sanitizer, n (%)	OR (95%CI)
**Age group**			
≤21	9 (16.4)	179 (31.2)	Ref
22–25	22 (40.0)	216 (37.6)	.71 (−.04–1.67)
≥26	24 (43.6)	179 (31.2)	.49 (.10–.99)
**Sex**			
Female	39 (70.9)	350 (61.0)	Ref
Male	16 (29.1)	224 (39.0)	−.45 (−1.13–.10)
**Education**			
No university entrance degree	11 (20.0)	36 (6.3)	Ref
University entrance degree	44 (80.0)	538 (93.7)	−1.32 (−2.09–−.49)
**Cases with vaccine status available**			
Cases not vaccinated against H1N1	46 (92.0)	352 (95.9)	Ref
Cases vaccinated against H1N1	4 (8.0)	15 (4.1)	.71 (−19.06–1.87)
**Cases diagnosed with H1N1**			
Cases not diagnosed with H1N1	50 (96.2)	370 (97.1)	Ref
Cases diagnosed with H1N1	2 (3.8)	11 (2.9)	.30 (−19.31–1.67)

### Cognitive and Affective Risk Perceptions: Descriptives and Mean Changes over Time

On average, participants estimated their personal likelihood of becoming H1N1-infected compared to a peer of the same sex and age as below average (see also Table 2). An optimistic bias in comparative numerical risk perceptions was evident at T1 (*M* = 3.4, *SD* = 1.2, *t*(628) = 11.9, *p*<.001) as well as at T2 (*M* = 3.7, *SD* = 1.0, *t*(428) = 6.0, *p*<.001). Likewise, estimations for the personal absolute likelihood of an H1N1 infection were significantly lower than the scale mean, with T1 (*M* = 2.3, *SD* = 1.2, *t*(628) = 33.7, *p*<.001) and T2 (*M* = 3.1, *SD* = 1.2, *t*(428) = 15.1, *p*<.001). In order to examine the mean level changes in both types of numerical-cognitive risk perceptions between T1 and T2, a 2×2 MANOVA for repeated measures was conducted, yielding a significant main effect for the factor ‘Time’, *F*(1, 428) = 135.4, *p*<.001 and Type of Risk Perception, *F*(1, 428) = 337.5, *p*<.001 which were qualified by a significant Time×Type of Risk Perception interaction, *F*(1, 428) = 48.9, *p*<.001. Subsequent analyses illustrated that participants showed a significant increase in both types of numerical-cognitive risk perception between T1 and T2, however, the increase was more pronounced in the perceived absolute likelihood, *F*(1, 428) = 160.9, *p*<.001 than in the perceived comparative likelihood of an H1N1 infection, *F*(1, 428) = 24.1, *p*<.001. With regards to the perceived severity of an H1N1 infection, at T1 participants perceived an H1N1 infection as being relatively serious, with a mean rating above the scale midpoint (*M* = 4.5, *SD* = 1.3, *t*(628) = 9.1, *p*<.001). Between T1 and T2, perceived seriousness decreased significantly, *F*(1,428) = 33.6, *p*<.001, with a mean rating of *M* = 4.1 (*SD* = 1.2) which was equivalent to the scale midpoint, *t*(428) = 1.1, *ns*.

**Table 2:** Intercorrelations and Descriptive Statistics for Numerical-Cognitive Risk Perceptions (Perceived Absolute and Comparative Likelihood, Perceived Severity) and Affect-Related Risk Perceptions (Perceived Threat, Concern, and Worry).

	2.	3.	4.	5.	6.	7.	8.	9.	10.	11.	12.	Range	*M*	*SD*
1. AbsoluteLikelihood T1	.42**	.44**	.30**	−.04	−.01	.37**	.29**	.42**	.30**	.33**	.27**	1–7	2.33 (2.28)	1.24 (1.21)
2. AbsoluteLikelihood T2		.34**	.47**	.02	.04	.29**	.25**	.30**	.35**	.24**	.21**	1–7	3.09	1.24
3. ComparativeLikelihood T1			.47**	.02	.00	.16**	.13**	.22**	.18**	.16**	.10*	1–7	3.42 (3.42)	1.22 (1.21)
4. ComparativeLikelihood T2				.02	.04	.11*	.08	.16**	.17**	.11*	.08	1–7	3.70	1.04
5. Severity T1					.37**	.25**	.11*	.29**	.20**	.27**	.16**	1–7	4.45 (4.50)	1.24 (1.30)
6. Severity T2						.10*	.15**	.18**	.25**	.10*	.26**	1–7	4.06	1.16
7. Threat T1							.53**	.75**	.52**	.70**	.44 **	1–4	1.47 (1.44)	0.70 (0.68)
8. Threat T2								.45**	.62**	.39**	.70**	1–4	1.36	0.59
9. Concern T1									.57**	.72**	.43**	1–7	2.19 (2.15)	1.27 (1.25)
10. Concern T2										.48**	.64**	1–7	2.05	1.07
11. Worry T1											.52**	1–4	1.78 (1.75)	0.78 (0.77)
12. Worry T2												1–4	1.60	0.66

*Note*.

* *p*<.05,

** *p*<.01;

correlations, *M*, *SD* for T1 are based on *N* = 629, T1 values in parentheses *n* = 429; T2 is based on *n* = 429.

In order to examine the mean level changes in the three different types of affective risk perceptions between T1 and T2, a 2×3 MANOVA for repeated measures was conducted, yielding a significant main effect for the factor ‘Time’, *F*(1, 428) = 10.8, *p*<.01 and ‘Type of Affective Risk Perception’, *F*(2, 856) = 265.0, *p*<.001. The ‘Time×Type of Affective Risk Perception’ interaction was not significant, *F* (2, 856)<1.8, *ns.* Subsequent analyses indicated that concern as well as perceived threat decreased significantly between T1 and T2, all *F's* (1, 428)>6.1, *p*<.05, whereas worry decreased only marginally, *F*(1, 428) = 3.04, *p* = .08.

### Who redeemed the voucher for a bottle of hand sanitizer and leaflets? Cognitive and affective risk perceptions predicting pick-up rates

In subsequent steps, the three proposed longitudinal models with the pick-up rate as dependent variable were tested: (1) the Parallel-Impact Model (M1), (2) the Time-Sequence Model (M2), and (3) the Dynamic Change Model (M3). Models were tested for the sample that filled in the first and second questionnaire (‘Sample T1–T2’, *n* = 429) and for the sample that filled in the first questionnaire (‘Sample T1’, *N* = 629).

#### Parallel-Impact Model (M1a–b)

In first step, the parallel impact of numerical-cognitive risk perceptions (perceived likelihood of infection, perceived severity, likelihood×severity interaction) and affect-related risk perceptions on the pick-up rate (T3) was examined. For Sample T1–T2 (*n* = 429), the analyses showed that affect-related risk perceptions (T2) predicted the pick-up rate at T3, with odds ratio [OR] = 1.23, 95% confidence interval [CI] = 1.11–1.37. Thus, the greater the negative affect associated with the new H1N1 influenza at T2 was, the more likely the participants were to pick-up the hand sanitary bottle and the leaflets at T3 (see also Table 3, Model 1a). Numerical-cognitive risk perceptions (T2), i.e. perceived likelihood and perceived severity of the new H1N1 influenza as well as their interaction, did not significantly predict the subsequent pick-up rate observed at T3. Additional analyses within Sample T1 (*N* = 629) using numerical-cognitive and affect-related risk perceptions measured at T1 as predictors for the pick-up rate at T3, did not yield statistically significant effects (see Table 3, Model 1b).

**Table 3:** Parallel Impact of Numerical-Cognitive Risk Perceptions (Perceived Likelihood and Severity) and Affect-Related Risk Perceptions (Index of Perceived Threat, Concern, and Worry) on Protective Behavior (T3).

	Affective risk perception at T2 (Regression coefficients)	Retrieval of hand sanitizer and leaflets at T3 (Odds Ratio, 95%-Confidence Interval; Regression coefficients for indirect effects)
*Model 1a (M1a): Parallel-impact*		*Direct effects (n = 429)*
Perceived likelihood T2	-	0.80 (.92–1.06)
Perceived severity T2	-	0.83 (.67–1.04)
Likelihood×severity T2	-	0.93 (.83–1.03)
Affective risk perception T2	-	1.23 (1.11–1.37)
*Model 1b (M1b): Parallel-impact*	-	*Direct effects (N = 629)*
Perceived likelihood T1	-	0.95 (.83–1.08)
Perceived severity T1	-	1.17 (.92–1.48)
Likelihood×severity T1	-	1.04 (.96–1.13)
Affective risk perception T1	-	1.04 (.94–1.15)

Results of logistic regression analysis.

#### Time-Sequence Model (M2a–c)

**Figure 1:**
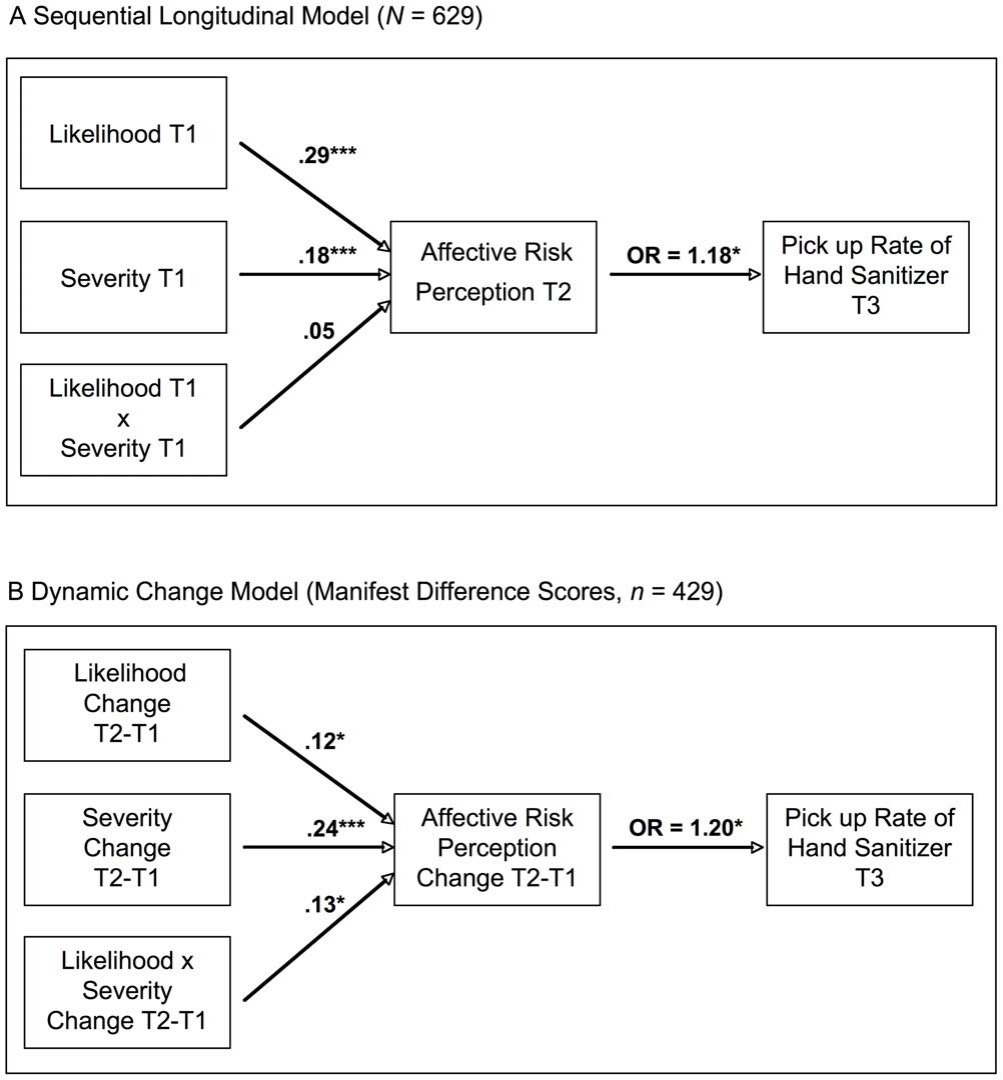
Static and dynamic change path models of the relationship between protective behavior (T3) and numerical-cognitive risk perceptions (perceived likelihood and severity) and affect-related risk perceptions (index of perceived threat, concern, and worry). *Note*. *p<.05, ***p<.001; standardized path coefficients are reported.

In a next step, the Time-Sequence Model was tested, assuming an indirect impact of numerical-cognitive risk perceptions (T1) on precautionary behavior (T3) through affect-related risk perception (T2). Analyses were conducted for Sample T1–T2 (*n* = 429). As shown in Figure 1a (see also Table 4, Model 2a), perceived likelihood (β = .29, *p*<.001) and perceived severity of an H1N1 infection at T1 (β = .18, *p*<.001) significantly predicted the amount of reported negative affect-related risk perception at T2. The more threatened, worried and concerned participants felt at T2, the more likely they were to redeem their voucher for a bottle of hand sanitizer at T3, with [OR] = 1.18, 95% CI = 1.07–1.30. For estimating the specific indirect effects for perceived likelihood and perceived severity, a non-parametric bootstrapping approach recommended by Preacher and Hayes [37] was employed. The bootstrapping approach yielded a significant indirect effect for both facets of numerical-cognitive risk perceptions, perceived likelihood, and perceived severity of an H1N1 infection at T1 on the pick-up rate at T3 through negative affect-related risk perception at T2 (see Table 3, Model 2a). Controlling for age and sex of the participants again yielded similar results (see Table 4, Model 2b).

**Table 4:** Sequential Impact of Numerical-Cognitive Risk Perceptions (Perceived Likelihood and Severity) and Affect-Related Risk Perceptions (Index of Perceived Threat, Concern, and Worry) on Protective Behavior (T3).

*Model 2a (M2a): Sequential-impact*	*Direct effects (n = 429)*	*Indirect effects (n = 429)*
Perceived likelihood T1	.29***	.07 (.02–.13)
Perceived severity T1	.18***	.07 (.02–.14)
Likelihood×severity T1	.05	.01 (−.02–.03)
		*Direct effect (n = 429)*
Affective risk perception T2	*-*	1.18 (1.07–1.30)
*Model 2b (M2b): Sequential-impact (controlling for age and sex)*	*Direct effects (n = 429)*	*Indirect effects (n = 429)*
Perceived likelihood T1	.25***	.06 (.02–.13)
Perceived severity T1	.26***	.06 (.02–.13)
Likelihood×severity T1	.12**	.01 (−0.01–.03)
		*Direct effect (N = 629)*
Affective risk perception T2	*-*	1.17 (1.06–1.29)
*Model 3a (M3a): Dynamic change model*	*Direct effects (n = 429)*	*Indirect effects (n = 429)*
Perceived likelihood T2-T1	.12*	.03 (.002–.08)
Perceived severity T2-T1	.24***	.09 (.03–.17)
Likelihood×severity T2-T1	.13*	.02 (.002–.05)
		*Direct effect (n = 429)*
Affective risk perception T2-T1	-	1.20 (1.06–1.36)
*Model 3b (M3b): Dynamic change model (controlling for age and sex)*	*Direct effects (n = 429)*	*Indirect effects (n = 429)*
Perceived likelihood T2-T1	.12*	.04 (.003–.10)
Perceived severity T2-T1	.24***	.10 (.04–.20)
Likelihood×severity T2-T1	.13*	.02 (.002–.06)
		*Direct effect (n = 429)*
Affective risk perception T2-T1	-	1.22 (1.07–1.40)

*Note*.

* *p*<.05,

*** *p*<.001.

Results of a path model with indirect effects of independent variables on precautionary behavior through affect-related risk perceptions and logistic regression of affect-related risk perceptions on precautionary behavior.

#### Dynamic Change Model (M3a–b)

In a final step, it was tested within Sample T1–T2 (*n* = 429) whether changes in numerical-cognitive risk perceptions were associated with changes in affect-related risk perception between T1 and T2 and whether changes in affect-related risk perception predicted the pick-up rate at T3. As shown in Figure 1b (see also Table 4, Model 3a), an increase in perceived likelihood (β = .12, *p*<.05) and perceived severity (β = .24, *p*<.001) and their interaction (β = .13, *p*<.05) was associated significantly with an increase in affect-related risk perceptions. Thus, a greater increase in perceived likelihood and severity of the H1N1 infection covaried with an increase in risk-related negative affect over time. Moreover, an increase in risk-related negative affect was in turn positivity related to a greater pick-up rate. Thus, when participants felt increasingly worried, concerned, and threatened by the H1N1 infection over time, they were more likely to come to the university office and to pick-up the hand sanitizer and leaflets. Again, controlling for the age and sex of the participants did not change the pattern of results substantially (see Table 4, Model 3b).

## Discussion

During a pandemic of the new strain of H1N1 influenza, the present prospective study examined two different types of perceived risk, numerical-cognitive and affect-related risk perceptions and their unique and joint influence on an objective indicator for self-protective behaviors, the redemption of a voucher for a free bottle of hand sanitizer and H1N1 information leaflets.

### Perceived Likelihood and Severity: Adaptive Changes over Time

The data show that participants' beliefs about the amount of risk they face changed over time. Between the first and second measurement point in time, perceived likelihood of an infection increased significantly and optimistic biases in risk perceptions decreased. At T2, participants believed that their own risk for becoming infected with H1N1 was nearly as high as the risk faced by an average peer. This is remarkable since numerous studies showed that people typically tend to believe that their own risk is lower than the risk of others [4], [6], [42]. Moreover, perceived severity of an H1N1 infection was quite substantial although few participants rated an H1N1 infection as life-threatening (less than 6%), concurring with others studies from the United Kingdom [19] and Australia [43]. Interestingly, perceived severity of the H1N1 infection decreased while perceived likelihood increased over time. One could argue that these changes in numerical-cognitive risk perceptions are relatively accurate reflecting the actual development of the pandemic situation at that time. Specifically, in October 2009 media coverage in Germany was at its peak since the public vaccination program was about to be started. Eight weeks later, more than 213,000 H1N1 infections in Germany were registered by the Robert Koch Institute but in most cases, the course of the infection was relatively mild. Thus, people might have had the experience that although more and more people became infected with the swine flu virus, even family members, friends or acquaintances, most people recovered quite quickly from the H1N1 infection after a few days of a sick leave. Van, McLaws, Crimmins, McIntyre and Seale [32] likewise argue that responses to a pandemic are subject to change in its pre-, early and mid-outbreak stages [19], [26].

### Setting the Stage for Protective Behavior: The Interplay of Cognitive and Affective Risk Perceptions

Extending previous research [17], [18], [20], the data showed a sequence of thoughts to feelings to protective behaviors within a context of a new pandemic: Greater numerical-cognitive perceived risk at T1 predicted greater affect-related risk perceptions at T2 which increased the pick-up rate at T3. This sequential longitudinal model was extended by a dynamic change perspective, demonstrating that changes in numerical-cognitive perceived risk between T1 and T2 covaried with changes in affect-related risk perceptions which predicted a greater pick-up rate. Moreover, the pattern of results remained virtually unchanged when controlling for age and gender. Thus, the data seem to be compatible with the ‘risk as feeling’ hypothesis that the health hazard triggered a sequence from rather “cold” numerical-cognitive risk perceptions to “hot” affect-related risk perceptions which in turn prompted protective behaviors.

Considered from a broader theoretical perspective, the concept of affect has received very little attention in research on the perception of health risks. Most studies examined the impact of numerical-cognitive aspects of risk perceptions on protective behaviors. However, various research findings show that the numbers of risk might be often perceived as a rather abstract information with only limited vividness and experiential value for most people [9], [44], [45]. Our results indicate that these cognitive, numerical representations of risk need to be translated into a more vivid, self-related and affect-related form of risk perception such as perceived threat, worry and concern in order to become motivationally relevant. Ditto et al. [44] also called this process ‘visceral motivation’ and Loewenstein et al. [9] conceptualized it as ‘risk as feeling’ (see also [46]). Abstract numbers of risk might therefore not impact protective behavior directly but trigger a more experiential or ‘visceral’ mode of risk perception such as perceived threat, concern, and worry which in turn increases protective behavior (cf., also [17], [20], [46]). Consistent with this notion, risk research has shown that generic risk information has limited impact on preventive behavior, whereas personalized risk feedback is more likely to trigger preventive intentions and behaviors [4]. The pursuit of these questions would be an important avenue for future research.

However, it is important to note that changes in cognitive-numerical risk perceptions and affect-related risk perceptions are empirically difficult to separate from each other because in reality people might not show one marked change in cognitive-numerical risk perceptions followed by a change in affect-related risk perceptions but they might rather show small changes which constantly feedback upon each other [9], [47]. Moreover, affective and cognitive evaluations of hazards may build upon different processing modes operating in parallel which feedback to another as suggested by dual processing models [48], [49]. Thus, it may be appropriate, at least in field studies, to conceptualize cognitive-numerical risk perception changes as either accompanying or preceding affect-related risk perception changes as indicators of a dynamic process. However since the current data are correlational, one cannot distinguish between these alternative possibilities definitely. In order to disentangle the sequence of changes in the two different types of risk perceptions and their impact on behavioral changes, an experimental design is needed in which both are independently manipulated (cf. also [17]). However, this remains a challenge for the future since experiments intended to alter risk perceptions seldom alter these perceptions substantially ([6], but see [15]) and are often unfeasible, due to ethical concerns, and lack the high ecological validity of the present design.

Moreover, one could alternatively argue that the data demonstrate a primacy-of-affect effect [50], affect-related risk perception being a direct and stronger predictor of behavior than numerical-cognitive risk perception. Accordingly, affect-related beliefs are a key to understanding protective behaviors, whereas numerical-cognitive risk perceptions might only be an epiphenomenon; and thus, affect-laden risk perceptions might be predominantly generated through other routes than numerical-cognitive risk perceptions [9], [10]. The primacy-of-affect hypothesis could explain why numerical-cognitive risk perceptions and the pick-up rate were not directly related. However, the missing direct link between numerical-cognitive risk perceptions and protective behavior might also be due to differences in past behavior [27]. The low numerical-cognitive risk perception - behavior relationship might be due to the fact that some people already use hand sanitizer. These people may believe that their infection-likelihood is very low (“I'm already cleaning my hand a lot, so my risk is low.”) but still have collected the hand sanitizer because they wanted a free refill. Thus, a confoundation in the observed pick-up rate between participants with a low infection likelihood perception because they already exhibit the behavior (accuracy hypothesis, [27]) and participants who had a high likelihood perception because they did not show the preventive behavior (motivational hypothesis) might have contributed to the low numerical-cognitive risk perception behavior relationship because these two opposing trends may have cancelled each other out.

### Limitations

Strengths of this study include the operationalization of self-protective behavior with an objective behavioral indicator (pick-up rate for a bottle of hand sanitizer and information leaflets). However, although the overall pick-up rate for the initial sample is rather low (8.7%), it is still comparable to other studies. National telephone surveys in the UK yielded a rate of 9.5% for sanitizing gel purchases in the UK [51]. A possible explanation for the low pick-up rate could be the course of the pandemic. In Germany, the first wave of the pandemic peaked in November 2009 with 37 fatal cases and almost 46,000 pandemic influenza cases in week 47 [30]–[31]. At the beginning of December 2009, the number of fatal cases and the infection rate decreased which might have rendered the use of hand sanitizers as rather dispensable in the eyes of the participants. However, the present data show that the perceived likelihood of infection was higher in December 2009 than in October 2009, suggesting that precautionary measures were still perceived as being a relevant behavior. This was also validated by the course of the pandemic since at the end of December 2009, the number of fatal cases began to increase again, and precautionary measures were still recommended by public authorities. The pick-up rate for a bottle of hand sanitizer is, in a strict sense, only a proxy for protective behavior and is, therefore, debatable. Whether it was actually used, was not assessed. The pick-up behavior itself was an intentional behavior which consumed significant time and effort since the participants needed to locate the office in the university building during certain opening hours. Since this behavior involved some planning and time resources, it seems plausible to assume that the hand sanitizer collected was actually used. However, whether it was used or not, was not assessed and it cannot be excluded that the hand sanitizer was simply stored by the participants or given away to others. Another clear limitation is that opportunistic studies, such as the present study, are prone to sampling biases. The vast majority of the participants had a high school degree, clearly limiting the generalizability of the findings. Moreover, the use of a snowball technique for recruiting participants could additionally limit the representativeness of the sample. In particular, it is likely that numerical literacy was comparably high, thus the degree to which the findings can be generalized is limited. The drop-out between T1 and T2 was 32%. Drop-out analyses did not show marked differences between the longitudinal sample and the drop-out sample in terms of perceived risk, sex, or education. However, participants may have drop-out of the study due to lack of interest in the flu topic. Since the present study focused on one preventive behavior, any generalizability regarding other preventive behaviors is limited. Increasingly negative affect-related risk perceptions may be effective in prompting certain preventive behaviors but might be ineffective in the context of other behaviors. Moreover, intervening to change one preventive behavior is likely to have knock-on effects for other behaviors. In a situation of severe pandemic flu, increasing affect-related risk perception may increase hand sanitization but it may also increase avoidance of travel and work, which may have much more deleterious effects on health than benefits gained through hand sanitization [21], [22]. Risk perception is only one out of an array of predictors for health behaviors according to current health behavior theories. Thus, explaining the whole variance observed in the respective behavior is not to be expected. Likewise, different protective behaviors may have very different determinants which behave in a dynamic manner as the epidemic unfolds thereby influencing a range of factors.

### Conclusion

In the present study, we examined in a longitudinal study of whether engaging in protective measures against the new H1N1 influenza is driven by numerical-cognitive and/or affect-related risk perceptions. We found that a perception of high likelihood and high severity of an influenza infection resulted in greater threat, concern, and worry; and that the more threatened, concerned and worried people were, the more likely they were to redeem the voucher. Therefore, we assume that perceived likelihood and severity of an influenza infection represent necessary but insufficient self-referential knowledge for paving the way for preventive behaviors. Affective components might be a necessary addition for models aiming at explaining health-protective behaviors.
